# Protective Effects of Methoxsalen Supplementation on Chronic Alcohol-induced Osteopenia and Steatosis in Rats

**DOI:** 10.3390/molecules25051177

**Published:** 2020-03-05

**Authors:** Ju Ri Ham, Ra-Yeong Choi, Hae-In Lee, Mi-Kyung Lee

**Affiliations:** 1 Department of Food and Nutrition, Sunchon National University, Suncheon 57922, Korea; punsu05@nate.com (J.R.H.); fkdud1304@naver.com (R.-Y.C.); 2 Mokpo Marin Food-Industry Research Center, Mokpo 58621, Korea

**Keywords:** alcohol, alendronate, bone quality, hepatosteatosis, methoxsalen, osteopenia

## Abstract

Osteopenia or osteoporosis occurs frequently in alcoholics and patients with alcoholic fatty liver disease. Methoxsalen (MTS), 8-methoxypsoralen, improved osteoporosis in ovariectomized and diabetic mouse models; however, its effects on alcohol-induced osteopenia and steatosis have not been reported. This study examined the effects of MTS on alcohol-induced bone loss and steatosis. Rats in the alcohol groups were fed a Liber-DeCarli liquid diet containing 36% of its calories as alcohol. MTS was at 0.005% in their diet, while alendronate (positive control; 500 μg/kg BW/day) was administered orally for eight weeks. The pair-fed group received the same volume of isocaloric liquid diet containing dextrin-maltose instead of alcohol as the alcohol control group consumed the previous day. In the alcohol-fed rats, the MTS and alendronate increased the bone volume density, bone surface density and trabecular number, while the bone specific surface, trabecular separation and structure model index were decreased in the tibia. MTS down-regulated tibial tartrate-resistant acid phosphatase 5 (*TRAP*) expression compared to the alcohol control group. MTS or alendronate prevented chronic alcohol-induced hepatic lipid accumulation and the triglyceride level in the alcohol-fed rats by decreasing the lipogenic enzyme activities and increasing the fatty acid oxidation enzyme activities. MTS reduced significantly the serum levels of alcohol, TRAP and tumor necrosis factor-α compared to the alcohol control group. Overall, these results suggest that MTS is likely to be an alternative agent for alcoholic osteopenia and hepatosteatosis.

## 1. Introduction

Chronic alcohol consumption-related diseases, including fatty liver disease, hepatitis, fibrosis and cirrhosis, are the leading causes of death for alcoholics [1]. Many studies have demonstrated that chronic and heavy alcohol intake is associated with impaired balanced bone remodeling and an increased risk of bone fragility [2]. Although the influence of alcohol on bone loss is controversial, alcohol-induced bone loss may eventually result in osteopenia, which has been attributed not only to the inhibition of bone synthesis but also to increased bone resorption through direct and indirect pathways [2]. Osteoporosis or osteopenia is a common complication of chronic liver disease, which is associated with age, BMI and alcohol liver disease [3]. Previous studies reported that patients with alcoholic liver diseases are more likely to have osteoporosis or osteopenia than those with chronic viral hepatitis [3,4]. Bisphosphonates and vitamin D supplementation have been used to prevent bone destruction and bone toxicity related to alcohol abuse in mice [5]. Many studies have examined natural plants and their bioactive compounds for preventing bone loss in the laboratory and in clinics [6,7].

Methoxsalen (8-methoxypsoralen, MTS), a natural photoactive compound, is found in various plants, such as celery, parsley, celeriac and lemon [8,9,10]. MTS is affiliated chemically to furanocoumarin, which has been used in combination with UVA irradiation as a photochemotherapeutic agent [11]. MTS has also been reported to have a range of physiological effects, including antioxidant [12], antiproliferative [13] and antimicrobial [14] activities. Moreover, MTS has anticancer effects, which increase the level of cytotoxicity in human hepatoma cells [15] and human osteosarcoma cells [16]. Previous studies showed that MTS has anti-osteoporosis effects in ovariectomized and diabetic mice [17,18], but the effects of MTS on alcoholic liver diseases have not been reported. Therefore, this study examined the efficacy and underlying mechanism of MTS on chronic alcohol-induced osteopenia and steatosis in rats.

## 2. Results

### 2.1. Bone Histomorphometric Parameters 

Chronic alcohol feeding caused a loss of body weights; however, both MTS and alenderonate (AD) did not affect the body weight and feed efficiency ratio (data not shown).

**Figure 1:**
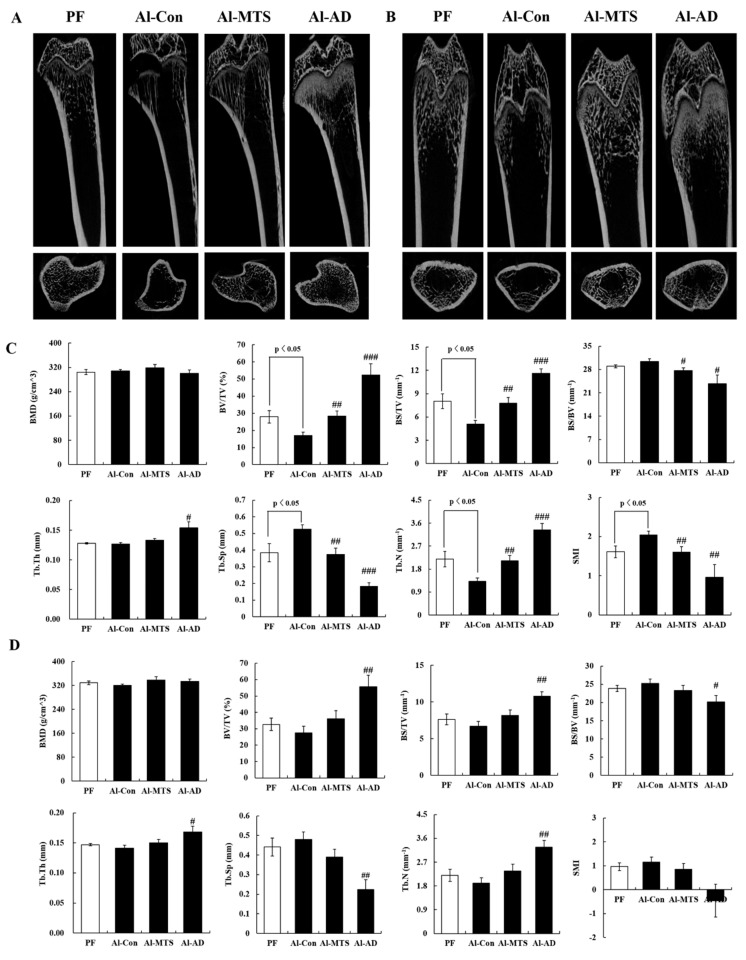
Effect of methoxsalen supplementation on the tibia micro-CT image (**A**), femur micro-CT image (**B**), tibia microarchitecture parameters (**C**) and femur microarchitecture parameters (**D**) in chronic alcohol-fed rats. The values are expressed as the mean ± S.E. # *p* < 0.05, ## *p* < 0.01 and ### *p* < 0.001 vs. Al-Con according to a Student’s *t*-test. PF; pair-fed, Al-Con: alcohol control, AL-MTS: alcohol supplemented with methoxsalen and Al-AD: alenderonate.

Two-dimensional images of the distal femur and tibia showed that eight-week alcohol (36% of total energy) feeding induced bone-loss (Figure 1A,B), but MTS and AD reversed it, which was more effective on the tibia than the femur (Figure 1). For the alcohol control (Al-Con) group, the tibial bone volume density (BV/TV), bone surface density (BS/TV) and trabecular number (Tb.N) were significantly lower than that in the pair-fed (PF) group, whereas trabecular separation (Tb.Sp) and structure model index (SMI) were significantly higher (Figure 1C). For the MTS and AD groups, the tibial BV/TV, BS/TV and Tb.N were significantly higher than the Al-Con group and Tb.Sp and SMI were significantly lower (Figure 1C). No significant difference in bone mineral density (BMD) was observed between the two groups (Figure 1). Although the femoral microarchitectures did not show significant changes by alcohol, AD increased BV/TV, BS/TV and Tb.N, while decreased Tb.Sp and SMI compared to the Al-Con group (Figure 1D).

### 2.2. Serum Bone Turnover Markers and Bone Remodeling-Related Gene Expression 

**Figure 2:**
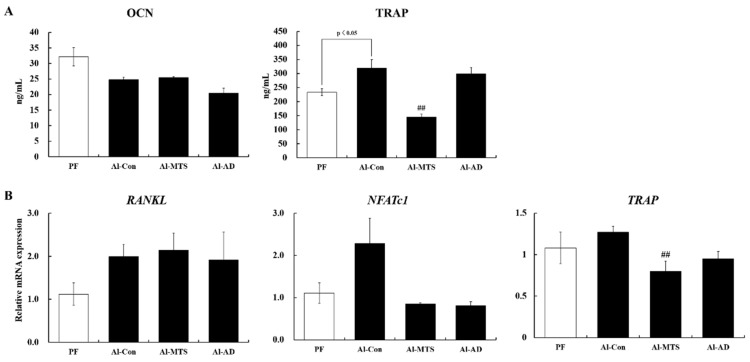
Effect of methoxsalen supplementation on serum osteocalcin (OCN) and tibial tartrate-resistant acid phosphatase 5 (TRAP) (**A**) and tibia osteoclast-related gene expression (**B**) in chronic alcohol-fed rats. The values are expressed as the mean ± S.E. ## *p* < 0.01 vs. Al-Con according to a Student’s *t*-test. *RANKL*: receptor activator of nuclear factor kappa-Β ligand and *NFATc1*: nuclear factor of activated T-cells, cytoplasmic 1.

The levels of bone formation marker, serum osteocalcin (OCN), were lower in the Al-Con group than in the PF group, but the decrease was not significant. The osteoclast differentiation-related factor, serum tartrate-resistant acid phosphatase 5 (TRAP) level, was significantly higher in the Al-Con group than in the PF group; however, MTS reduced the TRAP level significantly by 54.6%. AD did not alter serum the TRAP level (Figure 2A). The serum calcium (Ca) level was lower in the Al-Con group than in the PF group. MTS and AD did not affect the serum Ca and inorganic phosphorus (IP) levels (Table 1).

An upregulation trend in the gene expression of receptor activator of nuclear factor kappa-Β ligand (*RANKL*) and nuclear factor of activated T-cells, cytoplasmic 1 (*NFATc1*), by 1.7- and 2.1-folds, respectively, was observed in the tibia of the Al-Con group compared to the PF group. On the other hand, MTS and AD normalized the value of *NFATc1* expression (Figure 2B). In addition, MTS significantly down-regulated *TRAP* gene expression in the tibia compared to the Al-Con group (Figure 2B).

### 2.3. Hepatic Histology, Steatosis and Serum Alcohol Levels

**Figure 3:**
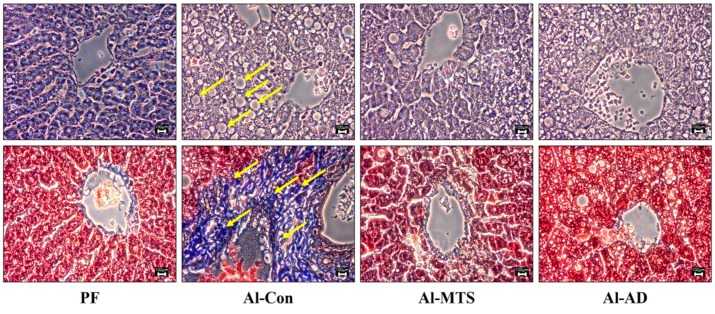
Effect of methoxsalen supplementation on hepatic hematoxylin and eosin and Masson’s Trichrome staining in chronic alcohol-fed rats. Magnification 400×; the yellow arrows indicate lipid droplets and inflammation.

As in Figure 3, hematoxylin and eosin (H&E) and Masson’s Trichrome staining showed that chronic alcohol intake caused the enlargement of hepatocytes, an increase in the number of lipid droplets and fibrosis. However, MTS and AD normalized the number and size of the lipid droplets, as well as the formation of collagen (Figure 3). Compared to the Al-Con group, MTS decreased significantly the hepatic triglyceride (TG) content (33.0%) increased by alcohol (Table 2).

The serum alcohol level was significantly higher in the Al-Con group than in the PF group but MTS and AD lowered it (Table 1). MTS and AD also tended to lower the serum acetaldehyde level by 57.6% and 65.6 %, respectively, compared to the Al-Con group (Table 1).

### 2.4. Serum Hepatotoxicity and Inflammatory Markers 

MTS and AD decreased the aspartate aminotransferase (AST; 48.0% and 14.7%, respectively) and alanine aminotranferase (ALT; 68.5% and 57.6%, respectively) levels compared to the Al-Con group, but the decrease was not significant (Table 1). The serum cytokines, such as tumor necrosis factor-α (TNF-α) and interleukin-6 (IL-6), were measured to identify the effects of MTS on the inflammatory response. The TNF-α level was increased significantly in the Al-Con group, but MTS and AD decreased it by 56.1% and 20.8%, respectively. The IL-6 levels were similar in all groups (Table 1).

### 2.5. Hepatic Alcohol and Lipid Metabolic Enzyme Activities 

Alcohol and MTS did not affect the hepatic alcohol dehydrogenase (ADH) and aldehyde dehydrogenase 2 (ALDH) activities, whereas AD decreased the ADH activity compared to the Al-Con group (Table 2). The cytochrome P450 2E1 (CYP2E1) activity in the Al-Con group was significantly higher relative to the PF group; however, MTS recovered to near the value of PF (Table 2).

The phosphatidate phosphohydrolase (PAP) activity in the Al-Con group was higher than that in the PF group. However, MTS suppressed the lipid synthetic enzyme activities, such as fatty acid synthase (FAS) and PAP, 37.6% and 34.7%, respectively) compared to the Al-Con group, while it increased the carnitine palmitoyltransferase (CPT) and fatty acid β-oxidation (β-oxidation) activity. AD only increased the β-oxidation activity (Table 2).

## 3. Discussion

The effects of alcohol intake on bone health are controversial, but many studies have reported that chronic alcohol consumption affects bone remodeling, resulting in bone loss and an increased risk of osteoporosis and fractures [19,20]. The present study also found that chronic alcohol feeding during eight weeks reduced the trabecular bone mass and impaired the bone microarchitecture, leading to osteopenia. The bone BMD is a major marker of quantity, while bone quality is defined as the bone material properties, such as cortical and trabecular microarchitecture, mineralization, turnover and collagen content and structure [21]. Herein, the serum alcohol concentration was not associated with the BMD, but it was negatively related to BV/TV, trabecular thickness (Tb.Th.) and Tb.N. and positively related to Tb.Sp and SMI (data not shown), indicating that rats chronically fed 36% of their dietary calories as alcohol showed a reduced trabecular number, bone volume and thickness. On the other hand, MTS supplementation (0.005% in diet) effectively recovered the alcohol-induced osteopenia and detrimental effects on the bone microarchitecture, which was similar to that of AD (bisphosphate drug against osteoporosis). AD is an aminobisphosphonate that inhibits bone resorption in osteoporotic human and rats [22]. In particular, the SMI is used to evaluate the characteristics of the rod and plate structure [23]. SMI in the Al-Con group was higher than PF, indicating that alcohol caused the formation of more rod structures, making them prone to fracture. However, both MTS and AD prevented the change in alcohol-induced rod-like structures in the tibia. Therefore, MTS prevented alcoholic osteopenia, as evidenced by the increased bone mass and bone quality.

The bone turnover markers have become an essential clinical tool for evaluating the bone structure [23]. In this study, MTS did not affect the serum OCN level (bone formation marker) but significantly lowered the serum TRAP (bone resorption marker) compared to the Al-Con group. TRAP is expressed in mature osteoclasts and plays a role in bone mineralization and skeletal development, indicating the amount and activity of osteoclast [24]. An increased TRAP level in the serum is associated with osteoporosis and other bone metabolic disorders [25]. Therefore, this study examined whether the protective effect of MTS against chronic alcohol-induced bone destruction is related to the changes in osteoclastogenesis-related genes. RANKL is an integral factor for osteoclast formation [26] and directly induced *NFATc1* expression, which stimulated RANKL-induced osteoclast differentiation [26]. Herein, *RANKL* and *NFATc1* expression had no statistical differences between Al-Con and PF, but alcohol led to a 1.7- and 2.1-fold increase in their expression, respectively, compared to PF. Iitsuka et al. [27] suggested that alcohol promoted osteoclastogenesis by increasing *RANKL* expression. *NFATc1* activates its target gene expression, such as *TRAP,* an osteoclast-specific sub-factor [28]. The present results showed that both MTS and AD similarly tend to down-regulate *NFATc1* and *TRAP* expression. In particular, MTS significantly suppressed *TRAP* expression, which led to a decrease in the serum TRAP level in chronic alcohol-fed rats. Therefore, the MTS-mediated decrease in TRAP is likely to be a key factor for improving the alcohol-induced osteopenia.

Alcoholic liver disease stimulates the release of cytokines, such as TNF-α, interleukin-1β (IL-1β) and IL-6, which promote the production RANKL and osteoclastogeneis [29]. A previous study showed that TNF-α is effectively accelerating RANKL production in osteoblasts, which increases osteoclasts formation [30]. This study found that chronic alcohol intake increases the serum TNF-α level compared to the PF group, but both MTS and AD effectively reversed these changes. Excessive alcohol consumption elevates the TNF-α level, which results in alcohol-related liver diseases, such as fatty liver, hepatitis, cirrhosis and cancer [31]. Fatty liver disease (steatosis) is a condition where there is more than 5% fat deposition in hepatocytes. Chronic alcohol consumption leads to an increase in the supply of lipids from the small intestine to the liver [32], mobilization of fatty acids from the adipose tissue [33] and cholesterol synthesis [34] and decreases fatty acid oxidation in the liver [35]. The present study showed that chronic alcohol consumption causes hepatic steatosis, as evidenced by the accumulation of hepatic TG and morphological changes (lipid droplets and fibrosis of hepatocytes), as well as an elevation of the serums AST and ALT. Therefore, this study examined the role of MTS on alcoholic steatosis by determining the alcohol and lipid metabolism in the liver. 

Alcohol is first metabolized to acetaldehyde and then to acetate [36]. Acetate converts to acetyl CoA, which can be used for energy or stored as a fat in the liver [37]. Excessive acetate leads to the accumulation of β-nicotinamide adenine dinucleotide and reduced disodium salt hydrate (NADH), which increases the level of fatty acid synthesis and TG deposition within the liver [38]. CYP2E1 can induce catalytic activity toward alcohol and generate a large amount of reactive oxygen species, which leads to alcoholic liver disease [38]. Therefore, reducing or suppressing CYP2E1 activity may be a practicable strategy for decreasing the hepatotoxicity of alcohol [39]. Lu et al. [40] examined whether CYP2E1 contributes to alcoholic fatty liver disease using CYP2El-knockout mice and suggested that CYP2E1-derived oxidative stress inhibits fatty acid oxidation, resulting in fatty liver disease. Our results also showed that the alcohol induced hepatic CYP2E1 activity and the accumulation of lipids, but MTS lowered the hepatic TG levels and lipid droplets significantly compared to the Al-Con group. A previous study reported that increased hepatic PAP activity was involved in the TG deposit in chronic alcohol-fed rats [41]. A similar change was observed in the present study. MTS significantly suppressed the FAS and PAP activities, which were increased by chronic alcohol intake. PAP and FAS are major enzymes in the TG and fatty acid synthesis pathways in the liver. On the other hand, hepatic CPT is a major enzyme of the fatty acid oxidation pathway. The inhibition of CPT leads to a decrease in fatty acid oxidation and a further increase in hepatic TG accumulation [42]. In this study, MTS elevated the level of fatty acid oxidation significantly in the liver of chronically alcohol-fed rats by stimulating CPT and β-oxidation. Interestingly, the hepatic TG concentration was positively correlated with the PAP activities and negatively correlated with CPT (data not shown). Therefore, MTS suppressed the lipogenesis (FAS and PAP) activities and increased the fatty acid oxidation (CPT and β-oxidation) activity, which may have helped to improve hepatic steatosis. 

## 4. Materials and Methods

### 4.1. Animals and Experimental Design

Sprague-Dawley rats (eight-week-old males) were purchased from Orient Bio Inc. (Seongnam, Korea) and housed individually under a 12-h light/12-h dark illumination cycle, 50% ± 5% humidity, and 20 ± 2 °C. The animals were used in accordance with the institutional guidelines, and the procedures were approved by the Institutional Animal Care and Use Committee of Sunchon National University (approval No. SCNU_IACUC-2018-03).

The animals were acclimatized for one week (chow and water ad libitum) and then divided randomly into the following four groups:

Group 1: pair-fed group (PF),
Group 2: alcohol control group (Al-Con),
Group 3: 0.005 % MTS (in diet) was supplemented with alcohol (Al-MTS) and
Group 4: alendronate (500 μg/kg BW/day, oral) was administered alcohol (Al-AD).

The alcohol-fed groups were introduced to an alcohol-containing liquid diet with a step-wise increase in alcohol: days 1 and 2, 3% alcohol (21% of the total energy); days 3 and 4, 4% alcohol (28% of the total energy) and after day 5, 5% alcohol (36% of the total energy). Group 1 was pair-fed and received the same volume of isocaloric liquid diet containing dextrin-maltose instead of alcohol as Group 2 consumed the previous day.

After eight weeks, the rats were anesthetized with CO_2_ gas after 12 h of food deprivation. Blood samples were then taken from the inferior vena cava. The serum was obtained by centrifuging the blood at 3000 rpm for 15 min at 4 °C. The organs (liver and bone tissues) were then removed, rinsed with physiological saline and weighed immediately.

### 4.2. Biomarkers In Serum 

The alcohol and acetaldehyde levels were determined using an enzymatic bioassay (Megazyme, Chicago, IL, USA). The OCN, TRAP, TNF-α and IL-6 levels were determined using a quantitative sandwich enzyme immunoassay (ELISA) kit (Elabscience Biotechnology Co., Ltd, Wuhan, China). AST, ALT, Ca and IP levels were measured using a diagnostic slide kit and an automatic analyzer (Dri-chem 3500i; Fujifilm Medical System Co., Ltd, Tokyo, Japan). 

### 4.3. Micro-Computed Tomography (Micro-CT) Assay 

After cleaning the adherent soft bone tissues and storing the bones in 70% ethyl alcohol, the femurs and tibias were analyzed using a high-resolution Skyscan micro-CT system (Skyscan 1272; Bruker, Billerica, MA, USA) and software as described previously [18]. The samples were scanned using a voxel size of 20.6 μm at 70 kV and 142 μA. Two-dimensional images were obtained for visualization and display. For morphometric analysis, the following structural parameters were calculated as a region of interest (ROI) of cancellous bone using CTAn (Bruker, Billerica, MA, USA). The BMD, BV/TV, BS/TV, bone specific surface (BS/BV), Tb.Th, Tb.Sp, Tb.N and SMI of each samples were then calculated. 

### 4.4. Quantitative Real-Time PCR Analysis in Bone 

The tibia was homogenized in TriZol reagent (Invitrogen, Carlsbad, CA, USA); after which, the total RNA was isolated according to the manufacturer’s specifications. The total RNA (1 μg) was then reverse-transcribed into cDNA using a ReverTra Ace qPCR RT master mix (Toyobo, Osaka, Japan). The level of mRNA expression was then quantified by real-time quantitative PCR using a SYBR green PCR kit (Qiagen, Hilden, Germany) and a CFX96TM real-time system (Bio-Rad, Hercules, CA, USA). The primer sequences are shown in the Supplementary File. The cycle thresholds were determined based on the SYBR green emission intensity during the exponential phase, and the fold changes were determined using the 2^−ΔΔCt^ method [43]. In addition, transcripts of *β-actin (Actb)* were amplified from the samples to ensure normalized real-time quantitative RT-PCR detection.

### 4.5. Histological and Lipid Contents Analysis in Liver 

For histological analysis, the liver was fixed in a buffer solution containing 10% formalin, processed routinely and embedded in paraffin. Blocks were cut to a 3–5-μm thickness, and sections were cut on glass slides and stained with H&E and Masson’s Trichrome. The stained area was then viewed using an optical microscope at 400× magnification. 

The hepatic lipids were extracted, as described previously [44]; after which, the TG, cholesterol (Asan Pharmaceutical Co., Ltd., Seoul, Korea) and free fatty acid (FFA) (Shinyang Diagnostics, Seoul, Korea) concentrations were determined using commercial kits.

### 4.6. Enzyme Activities in Liver

The following were determined as described previously: alcohol metabolic enzymes such as ADH, ALDH and CYP2E1 activities [41]; lipid metabolic enzymes activities such as FAS and PAP and β-oxidation and CPT activities [45].

### 4.7. Statistical Analysis

All data are presented as the means ± standard error (SE). Statistical analyses were conducted using the SPSS statistical software version 25.0 (SPSS Inc., Chicago, IL, USA). Comparisons between the groups were evaluated using a Student’s *t*-test. A *p*-value < 0.05 was considered significant. 

## 5. Conclusions

These findings are the first to show that MTS alleviates the chronic alcohol-induced deleterious changes in bone quality and steatosis through the modification of osteoclastogenesis and lipid metabolism. These features make MTS a promising candidate for the prevention of alcohol-induced bone loss and steatosis.

## Figures and Tables

**Table 1:** Effects of methoxsalen supplementation on serum parameters in chronic alcohol-fed rats.

	PF	Al-Con	Al-MTS	Al-AD
**Ca (U/L)**	10.01 ± 0.25	9.35 ± 0.14 *	9.71 ± 0.17	9.35 ± 0.24
**IP (U/L)**	8.14 ± 0.26	8.17 ± 0.26	7.80 ± 0.35	7.12 ± 0.45
**Alcohol (mg/L)**	33.75 ± 7.18	55.40 ± 5.09 *	30.27 ± 3.66 ^##^	28.12 ± 5.92 ^##^
**Acetaldehyde (mg/L)**	41.85 ± 11.62	66.57 ± 19.37	28.22 ± 5.03	22.90 ± 3.45
**AST (U/L)**	94.20 ± 9.34	374.80 ± 93.30 *	194.80 ± 24.63	319.83 ± 68.09
**ALT (U/L)**	30.00 ± 5.06	274.80 ± 87.44 *	86.60 ± 15.14	116.40 ± 18.14
**TNF-α (pg/mL)**	208.67 ± 23.43	299.62 ± 19.86 *	131.45 ± 34.99 ^##^	237.33 ± 9.74 ^#^
**IL-6 (pg/mL)**	4.33 ± 0.16	3.92 ± 0.17	3.56 ± 0.21	3.40 ± 0.09

Mean ± SE. * *p* < 0.05 vs. pair-fed (PF), ^#^ *p* < 0.05 and ^##^ *p* < 0.01 vs. alcohol control (Al-Con) according to a Student’s *t*-test. Al-MTS: alcohol supplemented with methoxsalen, Al-AD: alenderonate, Ca: calcium, IP: inorganic phosphorus, AST: aspartate aminotransferase, ALT: alanine aminotranferase, TNF-α: tumor necrosis factor-α and IL-6: interleukin-6.

**Table 2:** Effects of methoxsalen supplementation on hepatic lipid contents and metabolic enzyme activities in chronic alcohol-fed rats.

	PF	Al-Con	Al-MTS	Al-AD
**Hepatic lipid**				
**TG (mg/g)**	20.33 ± 1.33	30.01 ± 41.75 **	20.11 ± 0.84 ^##^	29.94 ± 0.94
**Cholesterol (mg/g)**	3.90 ± 0.15	5.29 ± 0.23 **	4.78 ± 0.44	4.96 ± 0.50
**FFA (mmol/g)**	9.30 ± 0.92	11.61 ± 0.70	10.25 ± 0.49	11.40 ± 0.63
**Enzyme activity**				
**ADH** ^1)^	384.77 ± 21.99	340.93 ± 8.61	347.45 ± 8.07	302.24 ± 15.16 ^#^
**ALDH** ^1)^	661.77 ± 24.08	671.40 ± 25.19	634.03 ± 43.12	620.51 ± 30.78
**CYP2E1** ^2)^	17.99 ± 0.53	22.04 ± 1.35 *	16.65 ± 1.03 ^#^	24.47 ± 1.35
**FAS** ^3)^	4.11 ± 0.15	4.36 ± 0.08	2.72 ± 0.21 ^###^	3.23 ± 0.35
**PAP** ^2)^	41.26 ± 3.45	69.48 ± 5.61 **	45.34 ± 4.15 ^#^	71.94 ± 16.92
**β-oxidation** ^2)^	1.33 ± 0.13	1.19 ± 0.10	1.57 ± 0.12 ^#^	1.55 ± 0.11 ^#^
**CPT** ^2)^	11.32 ± 0.82	10.87 ± 0.33	12.54 ± 0.51 ^#^	11.79 ± 0.81

Mean ± SE. * *p* < 0.05 and ** *p* < 0.01 vs. PF, ^#^ *p* < 0.05, ^##^ *p* < 0.01 and ^###^ *p* < 0.001 vs. Al-Con according to a Student’s *t*-test. ^1)^ pmol/min/mg protein, ^2)^ μmol/min/mg protein and ^3)^ nmol/min/mg protein. TG: triglyceride, FFA: free fatty acid, ADH: alcohol dehydrogenase, ALDH: aldehyde dehydrogenase 2, CYP2E1: cytochrome P450 2E1, FAS: fatty acid synthase, PAP: phosphatidate phosphohydrolase, CPT: carnitine palmitoyltransferase.

## Supplementary Materials

The following are available online.
