# Supplementary material for: Protective Effects of Methoxsalen Supplementation on Chronic Alcohol-induced Osteopenia and Steatosis in Rats
Source: Molecules. 2020 Mar 5;25(5):1177. doi: 10.3390/molecules25051177 (PMC7179412; doi:10.3390/molecules25051177)
Supplement: Supplementary file 1 [file molecules-25-01177-s001.pdf]

Table 1. Primer sequences for real time RT-PCR

Gene	Full name	Forward/Reverse(5'-3')
NFATc1 (NM_001244933.1)	Nuclear factor of activated T-cells	GCCCAAGTCTCTATCCCCAA/TCGATCCGGAGTTCATACGG
RANKL (NM_057149.1)	Receptor activator of nuclear factor kappa-B ligand	AAACAAGCCTTTCAAGGGGC/GAGCCACGAACCTTCCATCA
TRAP (NM_001270889.1)	Tartrate resistant acid phosphatase 5	TCTTCTACTGAGAGGTGCGAG/ATCACCAATCTCTCCCGTCC
Actb (NM_031144.3)	β-actin	ATGTGGATCAGCAAGCAGGA/AAAGGGTGTAAAACGCAGCTC
